# Corrosion-Resistant Steel–MgO Composites as Refractory Materials for Molten Aluminum Alloys

**DOI:** 10.3390/ma13214737

**Published:** 2020-10-23

**Authors:** Piotr Malczyk, Tilo Zienert, Florian Kerber, Christian Weigelt, Sven-Olaf Sauke, Hubertus Semrau, Christos G. Aneziris

**Affiliations:** 1 Institute of Ceramic, Glass and Construction Materials, TU Bergakademie Freiberg, Agricolastr. 17, 09599 Freiberg, Germany; tilo.zienert@ikgb.tu-freiberg.de (T.Z.); florian.kerber@ikgb.tu-freiberg.de (F.K.); christian.weigelt@ikgb.tu-freiberg.de (C.W.); aneziris@ikgb.tu-freiberg.de (C.G.A.); 2 ZPF GmbH, Petersaecher 4–6, 74963 Siegelsbach, Germany; sosauke@saukesemrau.de (S.-O.S.); prof_semrau@semrau.se (H.S.)

**Keywords:** metal matrix composites, molten aluminum, corrosion, wettability, surface oxidation

## Abstract

In this study, a novel metal matrix composite based on 60 vol% 316L stainless steel and 40 vol% MgO manufactured by powder metallurgy technology was developed. The corrosion resistance of the developed steel–MgO composite material against molten aluminum alloy AlSi7Mg0.3 was investigated by means of wettability tests and long-term crucible corrosion tests. The wettability tests were carried out using the sessile drop method with the capillary purification technique in a hot-stage microscope (HSM). Static corrosion tests were performed in molten aluminum alloy at 850 °C for 168 h to evaluate the impact of pre-oxidation of the composite surface on the corrosion resistance. The pre-oxidation of steel–MgO composites was carried out at 850 and 1000 °C for 24 h, based on preliminary investigations using thermogravimetry (TG) and dilatometry. The influence of the pre-oxidation on the composite structure, the corrosion resistance, and the phase formation at the interface between the steel–MgO composite and aluminum alloy was analyzed using SEM/EDS and XRD. The impact of the steel–MgO composite material on the composition of the aluminum alloy regarding the type, size, and quantity of the formed precipitations was investigated with the aid of ASPEX PSEM/AFA and SEM/EBSD. It was revealed that the pre-oxidation of the steel–MgO composite at 1000 °C induced the formation of stable MgO-FeO solid solutions on its surface, leading to a significant increase of long-term corrosion resistance against the liquid aluminum alloy.

## 1. Introduction

The chemical reactivity of molten aluminum alloys in contact with steel-based materials is well known and has already been widely studied [1,2,3,4]. A short contact time of steel with liquid aluminum is utilized for the formation of very robust, protective Fe-Al intermetallic layers on steel parts [5,6,7,8]. However, longer contact times of steel-based materials with liquid aluminum cause rapid dissolution of the steel and damage to the parts [2,4,9].

The wettability of metals and their alloys can be studied using various methods [10,11]. The wetting angle, which is measured between the molten metal and the substrate, is often used for the estimation of corrosion behavior or adhesion [11,12,13,14]. The wetting angle depends on the surface characteristics of the substrate, as well as on the composition and viscosity of the melt at a given temperature and atmosphere [15,16,17]. The viscosity of Al-Si alloys at casting temperatures is very low and decreases with the content of Si [16,18], entailing deep infiltration of the melt into the substrate and subsequent corrosion phenomena. The chemical interactions cause serious problems in the determination of aluminum wetting angles [13]. By means of sessile drop method with the capillary purification technique, the melt drop comes into contact with the substrate after reaching the melting point and the measurement is performed immediately after the deposition of the drop.

For the investigation of long-term corrosion in melts, static crucible tests or dynamic finger immersion tests are applied [19,20,21]. The immersion tests mostly take less than 12 h. The crucible corrosion test is commonly used in the field of refractory oxide ceramics and is characterized by significantly longer testing times [19,21,22]. For example, Sellers et al. [23] applied the crucible method to study the corrosion of 316L stainless steel and Hastelloy-N superalloy in a molten LiF-NaF-KF salt eutectic. No studies on the corrosion resistance of stainless steel against molten aluminum or aluminum alloy using long-term crucible tests were found in the literature search.

It is obvious that pure steel is unable to withstand even brief contact with molten aluminum [1,2,9]. Liquid aluminum alloy dissolves iron and forms complex phases in the ternary Al-Si-Fe system [3,24,25]. The dissolution of steel is continuously progressing as a function of time and does not omit other steel elements such as chromium or nickel [4,26,27,28,29]. Aluminum alloys also reduce most oxides [4,30,31]. Therefore, coatings applied on steel are sufficient only for a short period of contact with the melt [1,13,31,32].

Conventional refractory materials used for contact with molten aluminum and its alloys in different kind of furnaces are based on coarse-grained alumina–mullite castables with antiwetting agents to reduce the infiltration phenomena. The drying and prefiring processes used for the castables are very time-consuming and can cause internal stresses, leading to cracking and subsequent failure of the refractory product. Moreover, the thermal shock resistance of such castables is very often insufficient for the demanding, continuously changing thermal conditions of metallurgical operations [33,34].

Steel–ceramic composites benefit from the synergy between the steel and the refractory ceramics, and are characterized by good machinability, higher ductility, and advantageous thermomechanical properties, leading to better thermal shock resistance. The addition of ceramics to the steel can increase the corrosion resistance against molten aluminum alloy [35]. Furthermore, multiple studies in the field of steel–ceramic composites present their ability to be produced in a made-to-order fashion [36,37,38]. Additionally, their ability to form a reliable protective layer that can not be infiltrated and corroded by aluminum alloys makes steel–ceramic composites favorable substitutes for conventional refractory ceramics, especially for applications requiring refractory parts with sophisticated shapes, such as stirrers and lances. According to Fabrichnaya [39], magnesium oxide in contact with iron oxides forms complex MgO-FeO solid solutions or spinel structures based on MgFe_2_O_4_. This study focuses on the development of steel matrix composites reinforced by MgO and on the investigation of their corrosion resistance, particularly the contribution of an in situ formation of a passivated mixed oxide layer on the surface of the steel–MgO composite due to the pre-oxidation.

## 2. Materials and Methods

### 2.1. Materials and Manufacturing

The powder mixture used for manufacturing the composite samples consisted of 60 vol% gas-atomized 316L-FeCr18Ni10Mo3 stainless steel powder (TLS Technik, Bitterfeld-Wolfen, Germany) and 40 vol% electrofused MgO < 3 µm, 98% MgO (Refratechnik Steel, Duesseldorf, Germany). The mixture is further described as “316L–40MgO”. The composition of the steel powder is listed in Table 1. Table 2 contains the particle size percentiles and true densities of the raw materials.

Manufacturing of 316L–40MgO steel–ceramic composites requires a homogenous distribution of both steel and ceramic particles. For this purpose, the powder mixture was dry mixed on a roller mill for 120 min using 3 mm and 5 mm stainless steel mixing balls. The mass of the added balls was related to the mass of the powder mixture, which was equal to 21% and 16% for the 5 mm and for the 3 mm balls, respectively. After sieving out of the mixing balls, the Zusoplast WE52 liquid temporary additive (Zschimmer and Schwarz, Lahnstein, Germany) and water were stepwisely added to the powder mixture via granulation, ensuring a proper distribution of the binder and increasing the green body strength of the samples. The granulation procedure was carried out in two steps, first at 1600 min^−1^ for 2 min and subsequently at 2500 min^−1^ for 3 min, with the aid of an Eirich EL1 laboratory mixer (Maschinenfabrik Gustav Eirich, Hardheim, Germany).

The prepared composite granulates were pressed into cylindrical samples with the aid of a uniaxial press (Rucks, Glauchau, Germany). The pressing procedure had a consolidation pressure of 100 MPa preceded by two air degassing steps (30 MPa and 60 MPa for 1 s). For wettability tests, the samples with a diameter of 50 mm and height of 7 mm were used. The cylinders used for further preparation of crucibles had a diameter of 50 mm and a height of 50 mm.

After uniaxial pressing, all samples were dried in a convection drying oven. The samples used for wettability tests were dried at 110 °C for 24 h, whereas the cylinders used for preparation of crucibles were dried in two steps at 40 and 110 °C for 24 h each. After drying, a core bore with a diameter of ca. 36 mm and a depth of ca. 42 mm was hand drilled to prepare cylindrical crucibles with a wall thickness of 7–8 mm.

The binder removal was carried out in a debinding furnace (Xerion, Berlin, Germany) with heating rates of 2 K·min^−1^ to 200 °C and 0.5 K·min^−1^ from 200 to 500 °C, with a holding time of 30 min and cooling rate of 0.5 K·min^−1^. After debinding, the samples were sintered for 2 h at 1350 °C using a furnace with a graphite lining (Xerion, Berlin, Germany) under argon atmosphere, with heating and cooling rates of 5 K·min^−1^. The mean value of the linear sintering shrinkage was 4% and the bulk density was 4.76 g·cm^−3^. The measurement of the open porosity using an AutoPore V mercury intrusion porosimeter (Micromeritics, Unterschleißheim, Germany) revealed an open porosity of about 21%.

The wettability and corrosion resistance investigations were carried out using common silicon pre-eutectic AlSi7Mg0.3 casting aluminum alloy (TRIMET Aluminum, Essen, Germany). The as-delivered composition of this aluminum alloy is listed in Table 3.

### 2.2. Pre-Oxidation as a Surface Treatment

Both the wettability tests and crucible corrosion tests were performed on samples with two different surface treatments—as-sintered and pre-oxidized. The wettability substrate was pre-oxidized at 850 °C for 24 h. The steel–MgO composite crucibles for corrosion tests were pre-oxidized at 850 and 1000 °C for 24 h.

To evaluate suitable pre-oxidation conditions, thermal analyses of 316L–40MgO were performed using a STA 409 PC calorimeter (Netzsch, Selb, Germany) and a DIL 402 C dilatometer (Netzsch, Selb, Germany) under artificial air atmosphere conditions, with a heating rate of 10 K·min^−1^ up to 1100 °C. Moreover, to assess the oxidation kinetics, the thermogravimetric and dilatometric measurements were carried out for 24 h at constant temperatures of 850 and 1000 °C as functions of time.

### 2.3. Wettability Tests

As a necessary precondition for the measurement of wetting angles, a defined substrate surface is required. Therefore, the surfaces of the sintered substrates were stripped of the sintering oxide layer and polished with sandpapers of different grain sizes down to 8 µm. After polishing, one of the wettability substrates was pre-oxidized at 850 °C.

According to Wenzel [15], the equilibrium wetting angle *Θ*_E_ depends on the roughness of the substrate surface as follows:

(1) $$\Theta_{E}\;=\;\arccos\;\left(\frac{\cos\Theta_{W}}{S_{r}}\right)$$

where *Θ*_W_ is the apparent (as-measured) wetting angle and *S*_r_ is the developed area ratio (*S*_r_ = *Sdr* + 1) [40,41]. The linear or surficial roughness and the developed area ratio of the substrates were determined using a VK/X-1000 laser scanning microscope with a multifile analyzer (Keyence, Neu-Isenburg, Germany) according to ISO 25178-2-2020 and DIN EN ISO 4287 [40,42].

**Figure 1:**
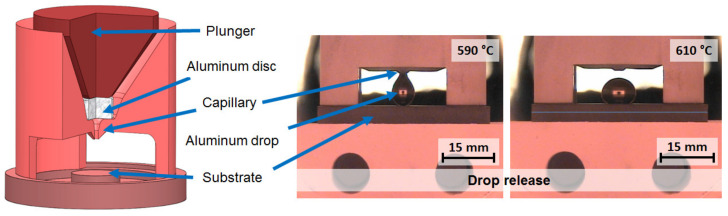
Design of the developed capillary purification system used for wettability tests (**left**) with exemplary images of drop release (**right**).

Wettability tests were performed using the sessile drop method with a capillary purification technique in a hot-stage microscope (HSM) (Raczek, Garbsen, Germany). For this purpose, a suitable capillary purification system was developed (see Figure 1). The capillary system with a pressurizing plunger was built from sintered, silica-free boron nitride with B_2_O_3_ binder (Henze Boron Nitride Products, Lauben, Germany). The capillary system was placed on top of the substrate. The aluminum alloy disc with a height of 3.5 mm and a diameter of 12 mm was put into the capillary chamber. The wettability tests were performed with a heating and cooling rate of 10 K·min^−1^ under argon atmosphere, with an oxygen level below 0.5 ppm. The contact angle was measured 30 s after the drop release at approximately 630 °C. Afterwards, the wetting angle between the substrate and the sessile drop was continuously recorded during heating up to 850 °C, with a holding time of 30 min at 850 °C, followed by cooling. An exemplary drop release process is shown in Figure 1.

After the wettability tests, the cross-sections of the substrates with solidified aluminum alloy were analyzed using energy-dispersive X-ray spectroscopy with the XL 30 scanning electron microscope (SEM/EDS) (Philips, Eindhoven, Germany).

### 2.4. Crucible Corrosion Tests

**Figure 2:**
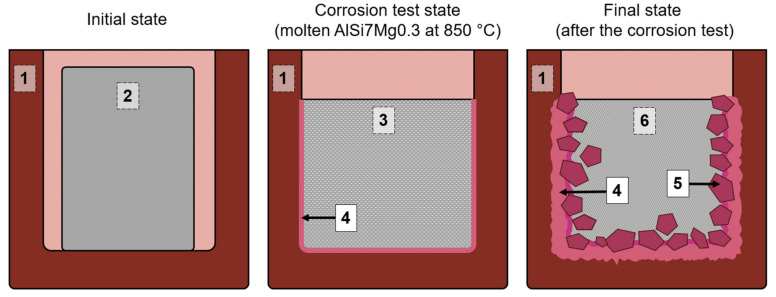
Schematic diagram of the crucible corrosion test: (1) crucible made from the steel–MgO composite, with an outer diameter of 50 mm; (2) solid aluminum alloy; (3) molten aluminum alloy; (4) crucible–aluminum alloy contact interface; (5) corrosion products; (6) solidified aluminum alloy after corrosion test.

The static crucible corrosion tests were performed in a laboratory furnace (Nabertherm, Bremen, Germany). For each crucible test, ca. 100 g of aluminum alloy AlSi7Mg0.3 was used. The crucibles with aluminum were heated up at 10 K·min^−1^ to 850 °C and held for 168 h (Figure 2). Subsequently, the samples with aluminum subjected to the corrosion test were vertically cut to analyze the cross-sections of the steel–ceramic composite, solidified aluminum, and particularly the interface between the composite and the aluminum alloy.

The microstructure analysis and the elemental mapping of the solidified aluminum alloy were carried out by SEM/EDS using the XL 30 (Philips, Eindhoven, Germany) and the ASPEX PSEM eXpress (FEI, Delmont, PA, USA) scanning electron microscopes.

X-ray diffraction (XRD) experiments were performed on polished crucible cross-sections with a Bragg–Brentano geometry and with Cu-K_α_ radiation between 25° and 100° 2Θ using the Empyrean DY1946 XRD diffractometer (Malvern Panalytical, Kassel, Germany). By using a divergence slit measuring 1/32°, only a very narrow area was irradiated (approximately 0.3 mm × 10 mm). The irradiation area was placed parallel to the composite crucible–aluminum alloy interface. Multiple scans at a distance of approximately 25 mm from the bottom of the crucible, with increments of 1 mm starting from the crucible side towards the aluminum alloy, were performed to identify and investigate the crucible–aluminum interface.

Phase analysis of the composite crucible was done using Rietveld analysis by applying the structure models listed in Table 4. For clarity, all XRD-detected phases were indicated based on their crystal structure. For example, the MgO- and FeO-related solid solutions, which are based on the NaCl structure, were named halite, instead of periclase and wustite.

The phase analysis for the aluminum alloy was performed using electron backscatter diffraction (EBSD) and SEM/EDS of the XL 30 (Philips, Eindhoven, Germany). Detected precipitations resulting from the reaction of aluminum alloy with the steel–MgO composite were indexed for further investigations.

**Figure 3:**
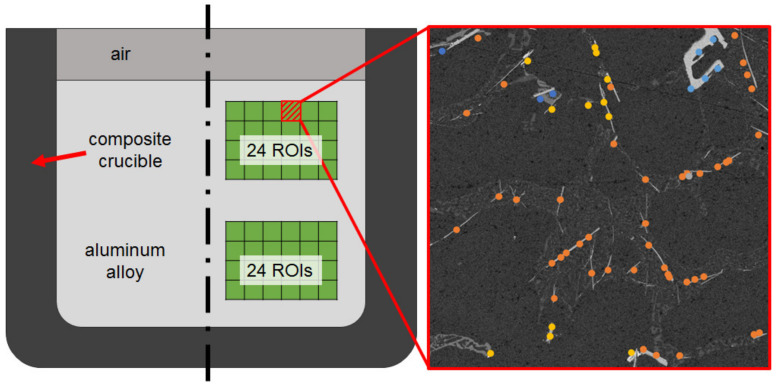
Schematic layout of regions of interest (ROIs) analyzed with ASPEX PSEM/AFA (**left**), with exemplary classification of detected precipitations within the solidified aluminum alloy (**right**).

The composition of the aluminum alloy and the assessment of which included the area fraction of the precipitated phases, was investigated by the ASPEX PSEM eXpress (FEI, Delmont, PA, USA) using automatic feature analysis (AFA). On each sample, a rectangular section with an area of at least 34 mm^2^ for the aluminum alloy was scanned and automatically divided into square regions of interest (ROIs) with edge lengths of ca. 111 µm. Corrosion phases were then identified by material contrast using the ASPEX PSEM back-scatter electron contrast. The detected precipitations were automatically analyzed by EDS and classified into indexed phases according to the restrictions of the elaborated rule file presented in Table 5. The quantification of the area fractions of indexed precipitations was based on the greyscale histogram analysis of 48 ASPEX PSEM/AFA ROIs. The positions of these ROIs within the solidified aluminum alloy and an exemplary phase classification procedure are schematically shown in Figure 3.

### 2.5. Sample Designation

To distinguish between the samples, the investigated samples were designated according to the type of test and temperature of the surface pre-oxidation. The designations W_0 and W_850 refer to the wettability samples with as-sintered and 850 °C pre-oxidized surfaces, respectively. The designations C_0, C_850, and C_1000 refer to crucible samples with as-sintered and with 850 °C and 1000 °C pre-oxidation surfaces, respectively.

## 3. Results and Discussion

### 3.1. Pre-Oxidation

**Figure 4:**
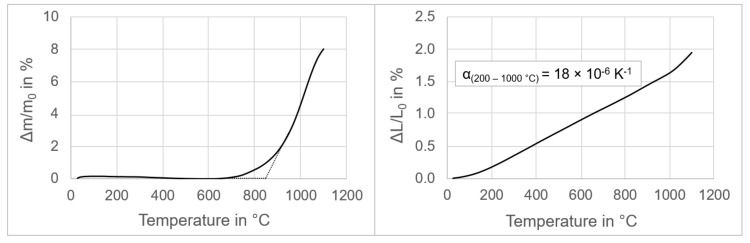
Thermogravimetry (**left**) and dilatometry (**right**) analyses of 316L–40MgO composite under air atmosphere as a function of temperature up to 1100 °C.

Pre-oxidation treatment was carried out to induce the formation of a passivation interface layer with increased corrosion resistance against molten aluminum alloy. The kinetics of the process, and hence the results of the pre-oxidation, are dependent on the temperature and duration of the oxidation. During the pre-oxidation, the samples undergo mass, dimensional, and microstructural changes. Figure 4 shows thermogravimetric and dilatometric results for the steel–MgO composite up to 1100 °C, with a heating rate of 10 K·min^−1^ under air atmosphere.

The composite showed no mass change up to 700 °C. The intersection point for the tangents belonging to the two linear sections of the thermogravimetric curve is at 850 °C. At 1000 °C, the sample already exhibited a mass increase of about 5%. Dilatometry analysis revealed constant thermal expansion of the composite from 200 °C to 1000 °C, with a linear thermal expansion coefficient of 18 × 10^−6^ K^−1^, i.e., similar to the thermal expansion coefficient of the 316L stainless steel specified in BS EN 10088-1:2014 [43].

**Figure 5:**
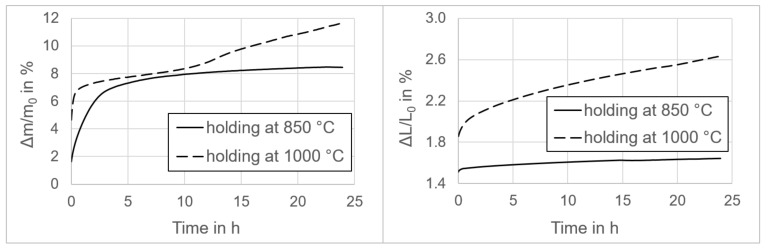
Isothermal thermogravimetry (**left**) and dilatometry (**right**) analyses of 316L–40MgO under air atmosphere as a function of time up to 24 h.

The long-term oxidation behavior of the composite as a function of time is shown in Figure 5.

The thermogravimetry analysis of the sample held at 850 °C proceeded as expected. The thermogravimetric curve had a logarithmic shape with rapid mass gain up to the 3rd hour. Until this time, the sample gained 7% of the initial mass. After 3 h, the mass gain significantly decreased and over the next 21 h the sample gained an additional 1.5% of the initial mass. The initial oxidation originated from the surface and continued through the open pores of the composite. Over the entire time range, the sample gained 8.5% of the initial mass.

The sample held at 1000 °C performed as expected up to the 10th hour, with a gain of 7% of the initial mass during the first 53 min of the experiment and exceeding 8.4% at the 10th hour. After this, the measurement revealed a sudden change of the curve slope, with a higher mass gain of about 0.2% per hour. This is probably related to the transformation of the surface structure of the composite, which acquired more oxygen from the atmosphere.

The dilatometry measurements revealed no unexpected thermal expansion behavior. After the 3rd hour of oxidation, both samples showed nearly linear expansion. The expansion rate of the sample held at 850 °C was ca. 3.5 × 10^−3^% × h^−1^ and after 24 h the sample gained 1.64% of the initial length. The expansion rate of the sample held at 1000 °C was ca. 23.8 × 10^−3^% × h^-1^ and after 24 h the sample gained 2.63% of the initial length.

### 3.2. Wettability Tests

As substrates with modified surfaces cannot be polished, their roughness needs to be determined experimentally. The linear and surficial characteristics and the developed area ratios for the W_850 and W_0 substrates are shown in Table 6.

**Figure 6:**
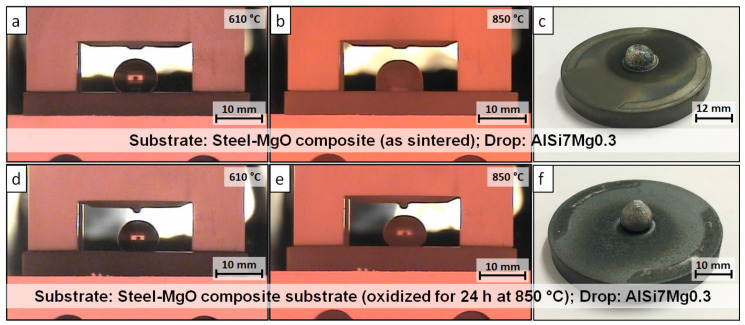
AlSi7Mg0.3 sessile drops on the 316L–40MgO composite substrates: (**a**) W_0 at the point of drop release; (**b**) W_0 after 30 min at 850 °C; (**c**) W_0 after the test; (**d**) W_850 after the drop release; (**e**) W_850 after 30 min at 850 °C; (**f**) W_850 after the test.

As expected, the pre-oxidation of the W_850 substrate resulted in increased roughness of the surface. The *S*_r_ parameter, which is necessary for the calculation of the equilibrium wetting angle, changed from 1.063 to 1.444. The wetting angles were measured 30 s after the drop release and after 30 min at a temperature of 850 °C. The results of the measurement with the corresponding drop images acquired during the experiments are shown in Figure 6 and Table 7.

The calculated equilibrium wetting angles of as-sintered and pre-oxidized samples show a clear difference. The wetting angle for W_0 was equal to 142.9°, whereas for W_850 was smaller and equal to 123.6°. It is assumed that a higher wetting angle results in reduced corrosion. However, the W_0 drop reacted with the substrate after 30 min at 850 °C. Due to the corrosion reaction, the wetting angle between the drop and the W_0 substrate could not be measured. The first visible reactions were observed by the change of the drop shape at 767 °C, i.e., approximately 14 min after the drop release. The W_850 sample showed a negligible change in the equilibrium wetting angle after 30 min at 850 °C. No visible reactions of the drop with the substrate could be observed.

**Figure 7:**
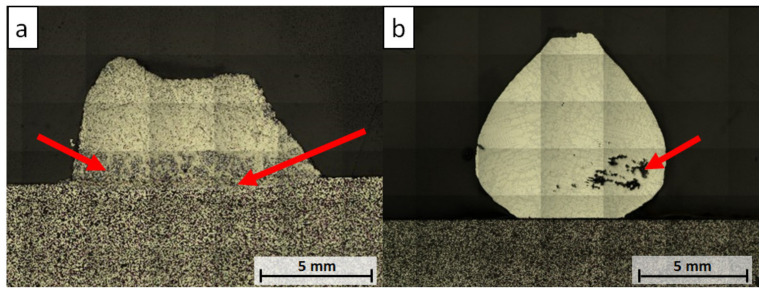
LSM image assembly micrographs of samples after wettability tests: (**a**) W_0; (**b**) W_850.

The overview of the samples’ cross-sections after the wettability tests was achieved using laser scanning microscope (LSM) image assembly and is shown in Figure 7.

The collapsed drop of the W_0 sample revealed vast corrosion, with visible corrosion products being observed on the polished cross-section with the bare eye. The aluminum melt diffused into the substrate and dissolved the steel matrix, forming multiple corrosion precipitations (see red arrows, Figure 7a). For the W_850 sample, no corrosion was detected using LSM. The drop porosity indicated by the red arrow (Figure 7b) was caused by the proceeding solidification of the aluminum alloy, which started from the surface of the drop and caused the formation of such cavities.

**Figure 8:**
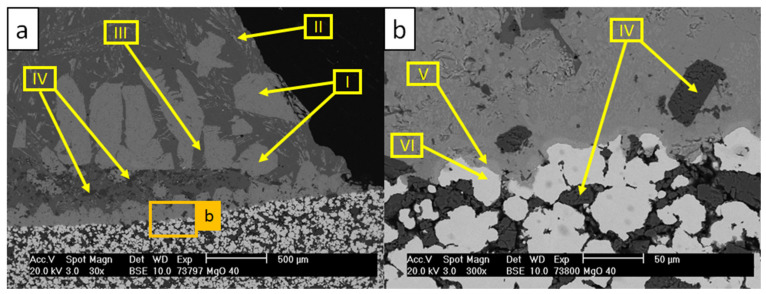
SEM micrographs of the W_0 sample after the corrosion test with AlSi7Mg0.3 at 850 °C: (**a**) image magnified to 30×; (**b**) image magnified to 300×.

Figure 8 and Table 8 show the microstructure of the W_0 sample and the corresponding results of EDS scans, respectively.

The micrographs in Figure 8 showed the corroded substrate with dissolved steel particles. As a consequence of the dissolution of the matrix material, MgO particles diffused into the aluminum alloy drop (scan IV). Multiple new formed phases were detected in the aluminum alloy. Precipitations marked with scan I were detected as τ_5_-Al(Fe,Cr)Si containing noticeable amounts of Cr [4,44]. A group of flake-like precipitations from scan II was identified as τ_6_-AlFeSi [3,4,45]. Scans V and VI represent a steel matrix–aluminum alloy interface, where the continuous dissolution of the substrate material was revealed.

**Figure 9:**
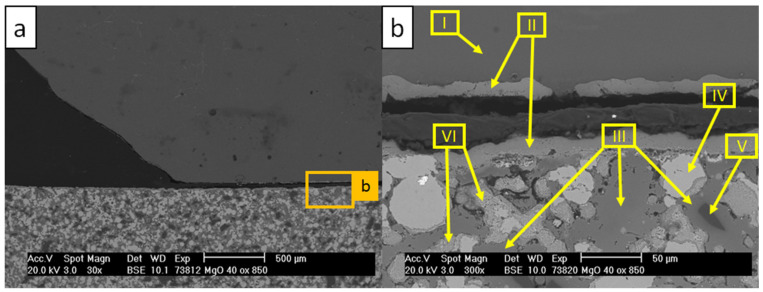
SEM micrographs of W_850 sample after the test with AlSi7Mg0.3 at 850 °C: (**a**) image magnified to 30×; (**b**) image magnified to 300×.

SEM analysis with the corresponding EDS scans of the W_850 sample are shown in Figure 9 and Table 9.

The micrographs revealed no corrosion of the substrate. Scans I and II represent the primary aluminum from the drop with the oxide residuals from the substrate surface. Under oxidizing atmosphere in contact with steel (scan IV), the MgO particles on the surface (scan V) transformed into Mg-Fe-O mixed oxides (scan III). The steel matrix partially oxidized (scans IV and VI). No dissolution of the oxide surface was observed during the wettability tests. Taking these observations into consideration, the surface of the 316L–40MgO composite after the 850 °C pre-oxidation can be described as resistant against the corrosion caused by the brief contact (approximately 30 min) with the liquid aluminum alloy.

### 3.3. Crucible Corrosion Tests

**Figure 10:**
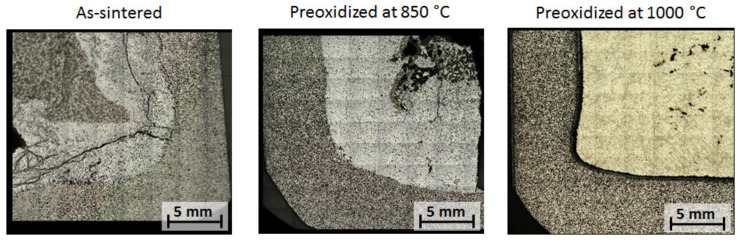
LSM image assembly micrographs of tested crucibles after 168 h contact with AlSi7Mg0.3 liquid aluminum alloy at 850 °C.

The LSM image assemblies of the cross-sections of tested crucibles are presented in Figure 10.

It was expected that the as-sintered composite crucible would dissolute over 168 h of contact with the liquid AlSi7Mg0.3, as the equivalent wettability substrates corroded and dissolved noticeably after 30 min contact with the alloy drop. Large fractures of the solidified alloy–crucible reaction zone were observed. During the dissolution of the matrix material, the MgO particles diffused into the melt and agglomerated in clusters (dark grey area in the melt). It is expected that the melt completely reacted with the steel and also reacted with the MgO particles, forming the Mg-Al spinel.

The C_850 crucible corroded, dissolving in the aluminum alloy. It is assumed that this proceeded through both the dissolution and infiltration of the passivated oxide surface. The aluminum melt infiltrating the crucible reacted with the steel particles, forming new phases under the oxide film. The new formed phases caused cracking of the passivated surface and facilitated further dissolution of the crucible.

The C_1000 crucible showed no damage after 168 h contact with the melt. Both the melt and the crucible remained separated after solidification. A common pre-eutectic micro structure of AlSi7Mg0.3 was observed in the LSM image assembly.

### 3.4. SEM and XRD Analysis of Composite Crucibles after Corrosion Tests

The microstructure of the composite crucibles after the corrosion test with aluminum alloy is presented below. The determination of the crucible structure by XRD was performed for the C_1000 sample.

**Figure 11:**
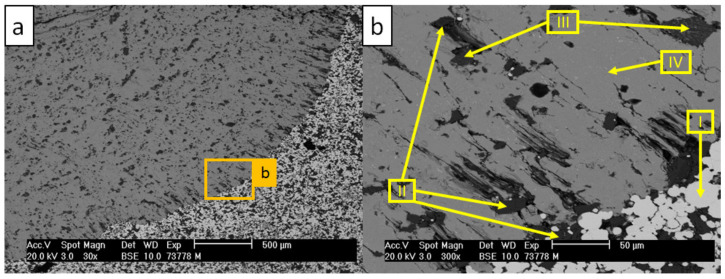
SEM micrographs of the C_0 composite crucible after the corrosion test with molten AlSi7Mg0.3 aluminum alloy at 850 °C: (**a**) image magnified to 30×; (**b**) image magnified to 300×.

Figure 11, along with Table 10, present the SEM analysis of the crucible–aluminum alloy interface of C_0, showing EDS scans of the most prominent phases.

Extensive dissolution of the steel matrix into the aluminum alloy was observed in the C_0 sample. The steel reacted with the aluminum alloy melt, forming large Al-Fe-Si solid solutions with a unified composition and a relatively high amount of Fe (scan IV). MgO particles reacted with Al residuals and formed an Al-Mg-O oxide mixture and MgAl_2_O_4_ spinel (scans II and III).

**Figure 12:**
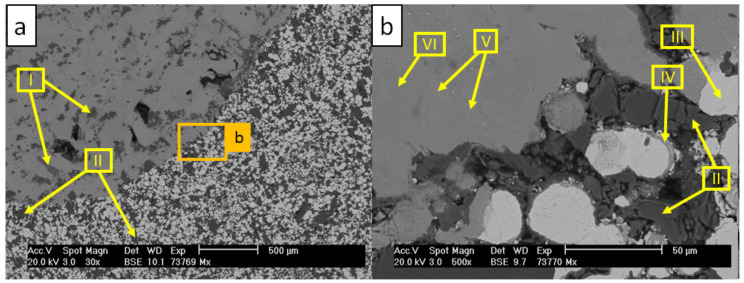
SEM micrographs of the C_850 composite crucible after the corrosion test with molten AlSi7Mg0.3 aluminum alloy at 850 °C: (**a**) image magnified to 30×; (**b**) image magnified to 500×.

Figure 12 and Table 11 present the SEM/EDS analysis of the C_850 crucible after the corrosion test at 850 °C.

**Figure 13:**
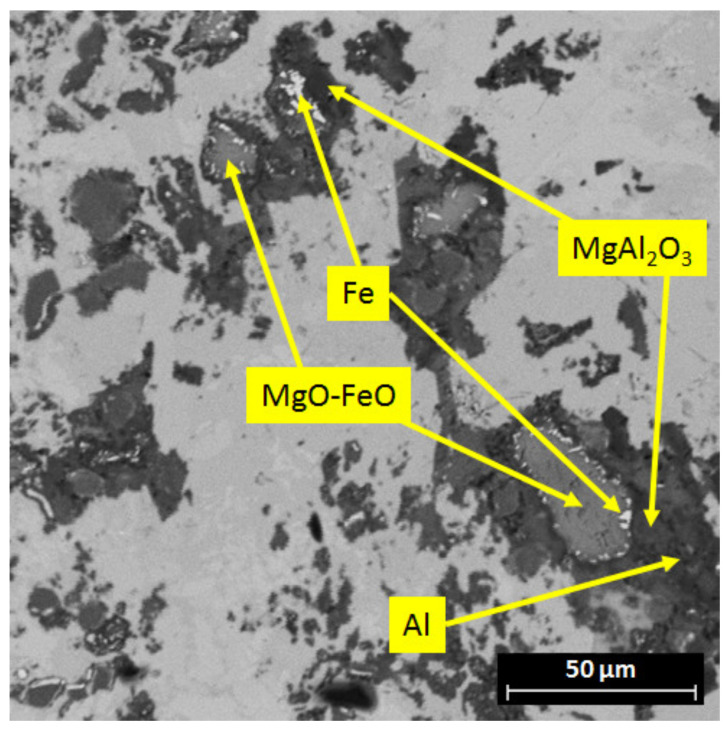
SEM micrograph of the C_850 sample with decomposition of the MgO-FeO solid solution to MgAl_2_O_4_ and Fe.

Similar to the C_0 sample, the C_850 crucible corroded greatly and its steel particles dissolved in the melt. The amounts of Fe and Cr in the compositions of the corrosion phases formed with aluminum (scans V and VI) were lower in comparison to the C_0 crucible (cf. Figure 11, scan IV). The MgO particles in contact with liquid aluminum underwent a transformation to Al-Mg-O mixed oxides (scans I and II). As was assumed, the MgO–FeO passivation layer was pushed out from the crucible vicinity during the corrosion process and was found in the upper part of the melt (Figure 13).

The residuals of the MgO-FeO crucible passivation layer reacted with aluminum and formed the MgAl_2_O_4_ spinel, reducing the Fe. The Fe precipitations could be found in the vicinity of the MgO-FeO solid solution and MgAl_2_O_4_. This revealed that the passivation surface of 850 °C pre-oxidized crucibles did undergo dissolution in contact with the liquid aluminum alloy.

**Figure 14:**
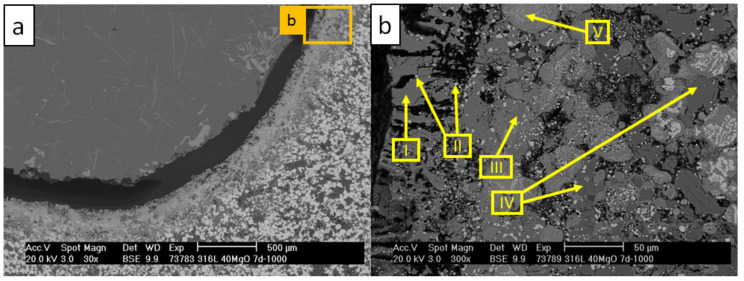
SEM micrographs of the C_1000 composite crucible after the corrosion test with molten AlSi7Mg0.3 aluminum alloy at 850 °C: (**a**) image magnified to 30×; (**b**) image magnified to 300×.

Figure 14 and Table 12 present the SEM/EDS analysis of C_1000 after 168 h contact with the AlSi7Mg0.3 melt.

**Figure 15:**
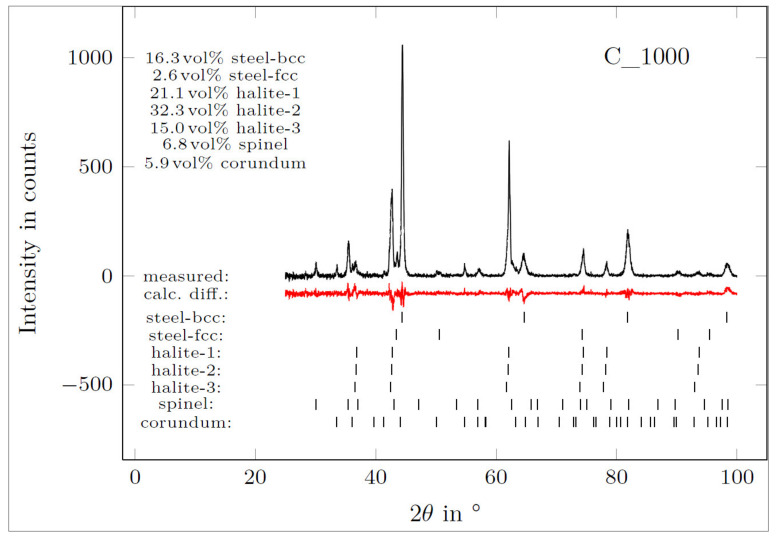
XRD pattern of the C_1000 crucible surface at the contact interface with aluminum alloy (detected phases designated based on their crystal structure).

No damage of the crucible passivation layer was revealed after 168 h contact with the melt. Only minor dissolution of the surface was observed, which contributed to the formation of Al-Fe-Si precipitations in the aluminum alloy in the vicinity of the crucible surface. The crucible revealed cavities at the crucible–aluminum alloy interface. These cavities, however, were not infiltrated by the aluminum alloy (scan I). Figure 15 and Table 13 show the diffraction pattern and results of XRD-phase analysis from the surface of that crucible.

**Figure 16:**
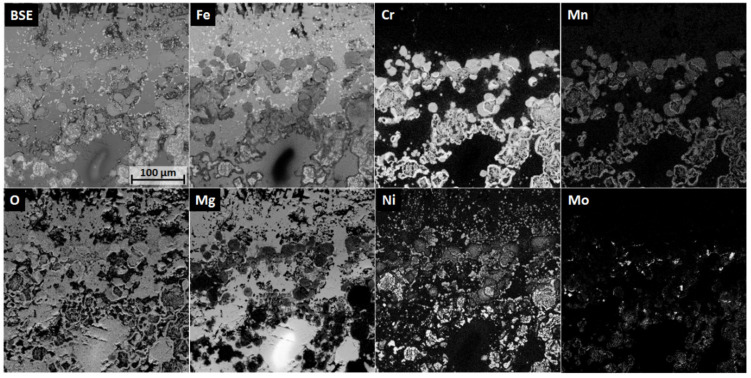
Detailed EDS elemental mapping at the aluminum alloy–steel–MgO composite interface of the C_1000 sample.

Multiple MgO-FeO solid solutions with differing compositions (halite structure, cf. Table 4) were found and are presented by scans I, III, and IV [46,47]. Three halite structures were found by XRD. Halite-1 corresponds with the MgO crystals, whereas halite-2 and halite-3 were MgO-FeO solid solutions with 11 and 25 vol% of FeO, respectively. It is assumed that halite-2 was caused by the MgO-FeO crystals growing epitaxially from MgO particles (scan IV) and that halite-3 was the MgO-FeO phase found near the aluminum alloy–crucible interface (scans I and III). The oxidized residuals of steel particles revealed Cr_2_O_3_ (corundum) and Fe_3_O_4_ (spinel) phases with metallic Ni (scan V) [48,49]. Among oxides, metallic Fe-Ni steel residuals were found (scan II). Only ca. 2.6 vol% of γ-Fe steel was found by XRD [50,51]. A detailed element mapping of aluminum–crucible contact surfaces collected over 48 h via ASPEX PSEM is presented in Figure 16.

No dissolution of the MgO-FeO solid solution passivation layer was observed. The Ni remained trapped in the Ni-Fe steel residuals and between the Cr_2_O_3_ and Fe_3_O_4_ mixture from oxidized steel particles. The crucible did not exhibit any damage from the contact with the liquid AlSi7Mg0.3. MgO particles reacting with steel during pre-oxidation at 1000 °C form a stable, homogenous passivation layer, which is resistant against long-term and high-temperature molten aluminum alloy corrosion.

### 3.5. Microstructure Analysis of AlSi7Mg0.3 after Crucible Corrosion Tests

The analysis of the aluminum alloy and the determination of the formed corrosion phases were performed on the C_1000 sample using SEM/EDS/EBSD and ASPEX PSEM/AFA methods.

**Figure 17:**
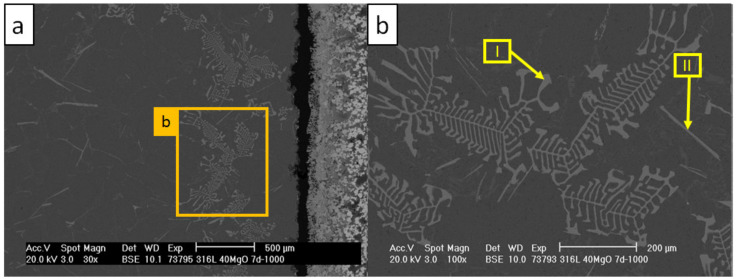
SEM micrographs revealing fishbone-like precipitations in AlSi7Mg0.3 close to the C_1000 interface: (**a**) image magnified to 30×; (**b**) image magnified to 100×.

**Figure 18:**
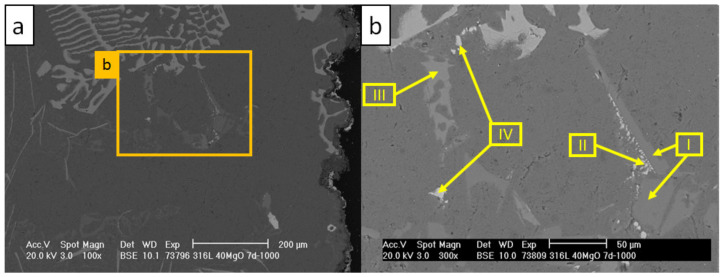
SEM micrographs revealing Si and Ni precipitations in AlSi7Mg0.3 in the vicinity of fishbone-like phases of C_1000: (**a**) image magnified to 100×; (**b**) image magnified to 300×.

The structures of the precipitating corrosion phases differed greatly depending on the distance from the crucible wall. Figure 17 and Figure 18 and Table 14 and Table 15 present the corrosion phases precipitated in the aluminum alloy at the contact boundary with the crucible.

**Figure 19:**
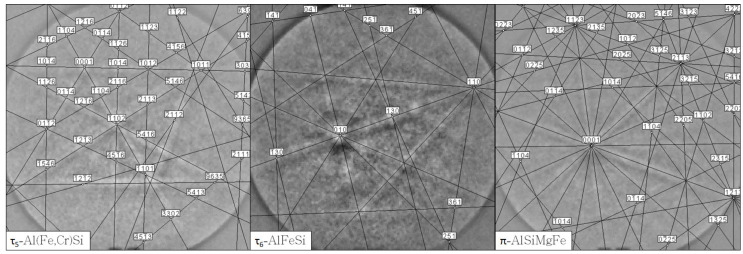
EBSD patterns of τ_5_-Al(Fe,Cr)Si and τ_6_-AlFeSi phases detected in the aluminum alloy of the C_1000 sample.

At the vicinity of the crucible wall, the fishbone-like precipitations (Figure 17, scan I) were revealed. These were indicated by EBSD as τ_5_-Al(Fe,Cr)Si phases based on the Al-Fe-Si ternary system [44]. The τ_6_-AlFeSi were found between the fishbone-like precipitations (Figure 17, scan II) [45]. In the vicinity of τ_5_, the Si- and Ni-rich precipitations (Figure 18, scans I and II, IV) and some π-AlSiMgFe phases were found (Figure 18, scan III) [24,52,53]. The indicated EBSD patterns of the indicated τ_5_, τ_6_, and π phases are presented in Figure 19.

**Figure 20:**
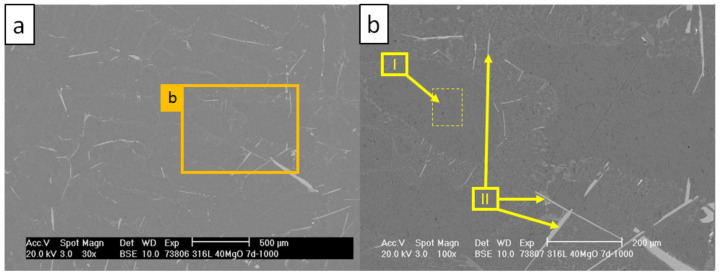
SEM micrographs revealing the AlSi7Mg0.3 microstructure at 1 mm distance from the C_1000 interface: (**a**) image magnified to 30×; (**b**) image magnified to 100×.

Figure 20 and Table 16 present the SEM/EDS results of the internal structure of the aluminum alloy collected at 1 mm distance from the interface of the C_1000 sample.

EDS and EBSD scans revealed the presence of multiple long (over 500 µm) τ_6_ precipitations, which were formed among the Al-Si eutectic (scan II). Scan I presents primary Al, which did not undergo any composition change. Moreover, no τ_5_ or other corrosion phases with higher amounts of Fe or Cr were found beyond 1 mm distance from the composite crucible.

The composition of the aluminum alloy of the C_1000 sample was analyzed by collective EDS scanning of the solidified aluminum alloy. In Table 17, the results of the scan and the initial AlSi7Mg0.3 composition are listed.

The amounts of steel- and MgO-related elements increased in relation to the total composition of the aluminum alloy by 0.5 wt %. The Fe amount increased by 0.7 wt %. The Ni and Cr amounts increased by 0.3 wt % and 0.2 wt %, respectively. It should be mentioned that the surface-area-to volume ratio (sa/vol) plays an important role in aluminum alloy composition changes. In the presented crucible corrosion tests, the aluminum–crucible contact surface area was roughly 51 cm^2^, whereas the volume of aluminum placed in the crucible was approximately 36 cm^3^. This gives a very high sa/vol ratio of about 1.42. In industrial environments, the casting furnaces are much larger, with an exemplary sa/vol ratio for the 1 m^3^ aluminum alloy equal to 0.05, which is 28.4 times smaller than the sa/vol ratio of the presented crucible corrosion experiment. It is obvious that the sa/vol ratio influences the density of the precipitating corrosion-related phases. For crucibles with industrial dimensions, the change of the alloy composition would be negligibly small.

The total number of corrosion-related phases precipitated in the aluminum alloy was calculated using ASPEX PSEM/AFA and is shown in Table 18.

The C_1000 sample had a total precipitation area fraction of 2.77%, which consisted mostly of fishbone-like τ_5_-Al(Fe,Cr)Si phases precipitated in the vicinity of the melt–crucible interface and τ_6_-AlFeSi phases in the middle of the melt. The sample exhibited 55.5% of τ_5_-Al(Fe,Cr)Si and 33.6% of τ_6_-AlFeSi phases. Additionally, 3.9% of π-AlSiMgFe and 5.9% of Ni-rich phases were found. Only 1.1% of all detected corrosion-related phases remained unclassified. The τ_5_ phases contained Cr in their structure. No Cr was found in the structures of the τ_6_ and π phases. The increased amounts of Cr and Ni in the solidified aluminum alloy indicate that the corrosion of the C_1000 crucible proceeded through dissolution of oxidized steel particles, which consisted of Cr_2_O_3_, Fe_3_O_4_, and metallic Ni-rich steel residuals. The low content of Mg-related π-AlSiMgFe phases is correlated with the stability of the MgO-FeO solid solution of that crucible, which did not dissolve over 168 h in contact with the melt. It can be stated that the corrosion of the C_1000 crucible proceeds through the dissolution of the oxidized residuals of steel particles from the vicinity of the composite–aluminum alloy interface. The diffusion of these oxides with trapped metallic Ni forms cavities in the crucible at the contact interface with the aluminum alloy (cf. Figure 14). No dissolution of the MgO-FeO solid solution at the crucible surface was observed for this sample. It is reasonable to presume that after the dissolution of the mentioned oxidized steel particles, the composite does not dissolve further and remains fully resistant against liquid aluminum alloy.

## 4. Conclusions

A novel 316L stainless steel + 40 vol% MgO (316L–40MgO) metal matrix composite was successfully developed and manufactured by means of powder metallurgy technology. The corrosion resistance of the composite against molten AlSi7Mg0.3 aluminum alloy at a temperature of 850 °C was evaluated. The investigation of the composite surface preparation revealed the favorable influence of the surface pre-oxidation on the corrosion resistance of the composite. The pre-oxidation causes the reaction between iron and MgO, forming MgO-FeO solid solutions, which are stable in contact with the AlSi7Mg0.3 melt.

It was revealed that the composites without surface oxidation were not corrosion-resistant in contact with the aluminum alloy melt and corroded rapidly. The corrosion was already observed after 814 min contact with the aluminum alloy drop during the wettability tests. The corrosion of the 316L–40MgO composite occurs mainly through the dissolution of steel particles forming corrosion phases from ternary Al-(Fe,Cr)-Si and quaternary Al-Si-Mg-Fe systems. Additionally, the MgO particles are not stable when in long-term contact with the liquid aluminum alloy and react to form a MgAl_2_O_4_ spinel.

The composite with its surface oxidized at 850 °C for 24 h exhibited short-term corrosion resistance against aluminum alloy, which could be observed during the wettability tests. Longer contact time with the aluminum alloy melt causes infiltration and damage of the passivation layer of the composite material.

Promising results were achieved for the composite with the surface pre-oxidized at 1000 °C for 24 h. This composite material did not reveal any damage after 168 h contact with the aluminum alloy melt. Partial dissolution of the oxidized steel particles at the interface with the liquid aluminum alloy was detected. These oxides, consisting of Cr_2_O_3_, Fe_3_O_4_, and Ni-Fe steel residuals, caused the precipitation of τ_5_-Al(Fe,Cr)Si, τ_6_-AlFeSi, π-AlSiMgFe, and local Ni-rich phases in the aluminum alloy. Nevertheless, the aluminum alloy composition revealed only minor changes and the area fraction of the precipitated phases was only 2.77%. Negligible dissolution of the MgO-FeO solid solution from the composite–aluminum alloy contact interface resulted in the precipitation of π-AlSiMgFe phases. It can be assumed that after initial dissolution, the 316L–40MgO composite remains fully resistant to the liquid aluminum alloy.

## Figures and Tables

**Table 1:** Composition of 316L stainless steel powder (in wt %).

Steel	Fe	Cr	Ni	Si	Mo	Mn	Ti	Nb	S	Al
316L	Balance	17.6	10.9	0.5	2.66	0.2	0.01	0.01	0.01	0.04

**Table 2:** Particle sizes and densities of the raw materials.

Raw Material	Particle Size in µm			Density in g·cm^−3^
	*D*_10_	*D*_50_	*D*_90_	
316L	4	30	53	7.94
MgO	0.7	14.5	67	3.60

**Table 3:** Composition of AlSi7Mg0.3 aluminum alloy (in wt %).

Alloy	Al	Si	Mg	Fe	Cu	Mn	Zn	Ti	Cr	Ni
AlSi7Mg0.3	92.30	7.17	0.27	0.081	0.002	0.002	0.007	0.12	0.001	0.003

**Table 4:** Overview of used structure models for the Rietveld refinement of 316L–40MgO/AlSi7Mg0.3 samples.

Structure Name	Corresponding Phases	Crystal System	ICSD
steel-fcc	γ-Fe	cubic	53449
steel-bcc	α-Fe solid solution	cubic	52258
halite	MgO, MgO-FeO solid solution	cubic	52026
corundum	Cr_2_O_3_	trigonal	25781
spinel	Fe_3_O_4_ solid solution	cubic	65341

**Table 5:** Rule file for the classification of precipitations in the AlSi7Mg0.3.

Class	Restrictions in wt %
AlSi7Mg0.3	Al > 70 AND Fe < 20
τ_6_-AlFeSi	Al > 20 AND Si > 1 AND Fe > 1 AND Cr < 1 AND Mg < 1 AND Ni < 5
τ_5_-Al(Fe,Cr)Si	Al > 20 AND Si > 1 AND Fe > 1 AND Cr > 1 AND Mg < 1 AND Ni < 5
π-AlSiMgFe	Al > 20 AND Si > 1 AND Fe > 1 AND Mg > 1 AND Ni < 5
Ni-rich phases	Ni > 5
Other precipitations	Balance

**Table 6:** Roughness characteristics of 316L–40MgO composite substrates according to ISO 25178-2-2020 and DIN EN ISO 4287 [40,42].

Sample	λc	Ra	Rz	Sa	Sz	Sr
-	-	µm	µm	µm	µm	-
W_0	0.8	1.14	9.06	1.28	57.27	1.063
W_850	2.5	3.56	31.15	3.78	67.62	1.444

**Table 7:** Wetting angles between AlSi7Mg0.3 and the 316L–40MgO composite substrate.

**Sample**	**Wetting Angle in °**			
**-**	**30 s after Release**		**after 30 min at 850 °C**	
**-**	***ϴ*_W_**	***ϴ*_E_**	***ϴ*_W_**	***ϴ*_E_**
W_0	148.0	142.9	-	-
W_850	143.0	123.6	142.0	123.1

**Table 8:** Results of EDS scans indicated by arrows in Figure 8 (in at %).

Scan	O	Al	Si	Fe	Cr	Ni	Mn	Mo	Mg
I	1.4	70.1	8.6	13.4	5.8	-	0.4	0.3	-
II	-	79.9	6.7	8.6	-	4.8	-	-	-
III	-	100	-	-	-	-	-	-	-
IV	53.3	1.0	-	-	-	-	-	-	45.7
V	-	76.4	5.2	13.9	2.7	1.6	0.2	-	-
VI	-	-	-	68.6	18.4	10.5	0.8	1.7	-

**Table 9:** Results of EDS scans indicated by arrows in Figure 9 (in at %).

Scan	O	Al	Si	Fe	Cr	Ni	Mn	Mo	Mg
I	-	100	-	-	-	-	-	-	-
II	69.0	-	-	26.4	-	-	-	-	4.6
III	58.5	-	-	11.4	0.2	-	0.6	-	29.3
IV	-	-	-	68.9	18.5	10.2	0.8	1.6	-
V	58.1	-	-	-	-	-	-	-	41.9
VI	52.7	-	0.4	24.0	13.5	4.2	0.7	0.9	3.6

**Table 10:** Results of EDS scans corresponding to areas indicated by arrows in Figure 11 (in at %).

Scan	O	Al	Si	Fe	Cr	Ni	Mn	Mo	Mg	Ti	Na	Ca
I	-	-	0.2	65.1	21.9	9.6	2.1	1.1	-	-	-	-
II	35.9	-	-	0.4	0.6	0.1	1.2	-	60.6	-	1.2	-
III	39.8	39.7	0.2	0.5	0.2	0.3	0.1	-	18.3	-	0.4	0.5
IV	1.2	60.9	4.1	20.7	6.8	4.8	0.7	0.2	0.5	0.1	-	-

**Table 11:** Results of EDS scans corresponding with areas indicated by arrows in Figure 12 (in at %).

Scan	O	Al	Si	Fe	Cr	Ni	Mn	Mo	Mg	Na	Ca
I	39.0	41.9	0.6	0.1	0.1	0.3	0.1	-	17.4	0.4	0.1
II	53.5	-	-	-	-	-	-	-	46.5	-	-
III	-	-	0.5	68.2	18.3	10.2	1.0	1.8	-	-	-
IV	65.0	-	0.4	8.2	15.1	1.1	2.7	0.7	7.0	-	-
V	-	76.1	3.3	16.5	2.2	1.9	-	-	-	-	-
VI	-	77.7	7.0	12.1	2.3	0.9	-	-	-	-	-

**Table 12:** Results of EDS scans corresponding with areas indicated by arrows in Figure 14 (in at %).

Scan	O	Al	Si	Fe	Cr	Ni	Mn	Mo	Mg
I	59.1	-	-	24.9	-	-	0.8	-	15.2
II	0.4	0.2	0.2	75.3	1.1	22.6	-	0.2	-
III	57.0	-	-	23.9	0.6	0.8	0.7	-	17.0
IV	57.0	-	-	12.7	0.6	0.3	0.3	-	29.1
V	61.5	-	-	19.0	10.6	4.5	-	-	4.4

**Table 13:** Phases detected at the surface of the C_1000 sample (designated based on their crystal structure).

Sample	Structure Name	Corresponding Phases	Density in g·cm^−3^	vol%	Lattice Parameter
C_1d_1000	halite-1	MgO	3.56	21.1	*a* = 4.21923 Å
-	halite-2	MgO-FeO solid solution	3.54	32.3	*a* = 4.22753 Å
-	halite-3	MgO-FeO solid solution	3.49	15.0	*a* = 4.24678 Å
-	steel-bcc	γ-Fe	7.74	16.3	*a* = 2.88222 Å
-	steel-fcc	α-Fe solid solution	7.94	2.6	*a* = 3.60186 Å
-	spinel	Fe_3_O_4_ solid solution	5.22	6.8	*a* = 8.38222 Å
-	corundum	Cr_2_O_3_	5.21	5.9	*a* = 4.96854 Å *c* = 13.59768 Å

**Table 14:** Results of EDS scans corresponding with areas indicated by arrows in Figure 17 (in at %).

Scan	Mg	Al	Si	Cr	Fe	Ni
I	-	75.9	10.8	3.5	9.8	-
II	0.4	68.9	12.1	0.3	17.8	0.5

**Table 15:** Results of EDS scans corresponding with areas indicated by arrows in Figure 18 (in at %).

Scan	Mg	Al	Si	Fe	Ni
I	-	4.5	95.5	-	-
II	1.4	79.7	12.4	-	6.5
III	23.1	45.7	26.3	2.1	2.9
IV	6.5	57.6	15.7	-	20.2

**Table 16:** Results of EDS scans corresponding with areas indicated by arrows in Figure 20 (in at %).

**Scan**	**Mg**	**Al**	**Si**	**Fe**
I	0.9	97.1	2.0	-
II	-	69.1	17.9	13.0

**Table 17:** Composition of AlSi7Mg0.3 as delivered and after corrosion tests with C_1000 (in wt %).

**Aluminum Alloy**	**Al**	**Si**	**Mg**	**Fe**	**Cu**	**Mn**	**Zn**	**Ti**	**Cr**	**Ni**
as delivered	92.30	7.17	0.27	0.081	0.002	0.002	0.007	0.12	0.001	0.003
after corrosion test with C_1000	89.63	6.55	0.70	0.77	-	0.08	-	0.10	0.20	0.30

**Table 18:** Area fraction and proportion of corrosion-related phases precipitated in AlSi7Mg0.3.

Sample	Area Fraction of Precipitations (in %)	Precipitated Corrosion-Related Phases (in %)				
		τ_6_-AlSiFe	τ_5_-Al(Fe,Cr)Fe	π-AlSiMgFe	Ni-rich	others
C_1000	2.77	33.6	55.5	3.9	5.9	1.1
